# Self-reported near-death and near-death-like experiences in a Chinese online sample: an exploratory mixed-methods study

**DOI:** 10.3389/fpsyg.2026.1935246

**Published:** 2026-09-21

**Authors:** Yufei Xue, Fangting Wang, Chunyan Yang, Runzhi Zhang, Huiyu Zou, Yiyun Zhang, Steven Laureys, Haibo Di

**Affiliations:** 1 International Unresponsive Wakefulness Syndrome and Consciousness Science Institute, School of Basic Medicine, Hangzhou Normal University, Hangzhou, China; 2 School of Basic Medical Sciences, Hangzhou Normal University, Hangzhou, China; 3 Zhejiang-Belgium Joint Laboratory for Disorders of Consciousness, Hangzhou Normal University, Hangzhou, China; 4 School of Public Health and Nursing, Hangzhou Normal University, Hangzhou, China; 5 School of Clinical Medicine, Hangzhou Normal University, Hangzhou, China; 6 Joint International Research Unit on Consciousness, CERVO Brain Research Centre, University of Laval, Québec City, QC, Canada

**Keywords:** Chinese culture, consciousness, mixed-methods study, NDE-C scale, near-death experiences, near-death-like experiences, thematic analysis

## Abstract

**Introduction:**

Near-death experiences (NDEs) are subjective experiences reported during actual or perceived life-threatening situations, whereas near-death-like experiences may occur in non-life-threatening altered states. Empirical data from Chinese populations remain limited, particularly regarding culturally specific narrative content.

**Methods:**

This exploratory mixed-methods study examined self-reported NDEs and NDE-like experiences in a Chinese online convenience sample. Participants recruited through Chinese social media completed an online survey comprising sociodemographic questions, the Chinese Near-Death Experience Content (NDE-C) scale, and an open-ended narrative question. Quantitative analysis included 63 participants: 23 NDE cases, 25 NDE-like cases, and 15 below-threshold comparison cases. All classifications were based on self-report. Qualitative thematic analysis was conducted on 48 valid narratives.

**Results:**

Self-reported NDE and NDE-like narratives shared recurrent features, including out-of-body experiences, bright light, bodily sensations, emotional changes, and altered perceptual content. Compared with NDE-like narratives, NDE narratives showed descriptively higher frequencies of peaceful emotions, encounters with entities, deceased loved ones or spiritual entities, personal insights, panoramic life review, and culturally specific Chinese imagery. Chinese cultural motifs were infrequent overall, occurring in four of 23 NDE narratives and one of 25 NDE-like narratives. These motifs included Guimen Pass, Black and White Impermanence, Lord Chenghuang, reincarnation, karmic judgment, and underworld-related imagery.

**Discussion:**

Because this study relied on retrospective self-report, an online convenience sample, and unverified medical contexts, the findings should be considered preliminary and hypothesis-generating. The findings provide localized exploratory evidence that Chinese self-reported NDE narratives may combine recurrent NDE-related features with culturally specific symbolic content.

## 1 Introduction

Near-death experiences (NDEs) are commonly described as subjective experiences occurring during actual or perceived life-threatening situations, often involving out-of-body experiences, altered time perception, tunnel-like sensations, bright light, encounters with deceased persons or other entities, life review, and intense emotional states (Martial et al., 2020a; Angeli-Faez et al., 2025). In contemporary consciousness research, NDEs have increasingly been discussed as a distinctive form of altered consciousness in which rich internal awareness may occur despite reduced behavioral responsiveness and limited connectedness with the external environment (Martial et al., 2020a; Peinkhofer et al., 2021). This view is consistent with multidimensional models of consciousness that distinguish wakefulness, internal awareness, and environmental connectedness as partially separable dimensions (Martial et al., 2020a). From this perspective, NDEs are not merely unusual memories or retrospective stories, but potentially important subjective reports for examining how consciousness, self-related experience, and meaning-making may persist or emerge under extreme physiological and psychological conditions (Martial et al., 2020a; Angeli-Faez et al., 2025).

Near-death-like experiences (NDE-like experiences) share some phenomenological features with NDEs but occur in contexts that are not necessarily life-threatening, such as syncope, sleep-related states, deep meditation, or other altered states of consciousness (Charland-Verville et al., 2014; Cassol et al., 2018; Pehlivanova et al., 2023; Woollacott et al., 2024). Previous research has suggested that NDEs and NDE-like experiences may overlap in elements such as out-of-body perception, bright light, bodily detachment, a sense of peace, and ineffability, although they may differ in trigger, narrative coherence, perceived reality, and personal significance (Charland-Verville et al., 2014; Cassol et al., 2018). Recent within-subject comparisons of near-death and psychedelic experiences have further shown that different altered states may share phenomenological features while still differing in perceived reality, interpretation, and enduring effects (Martial et al., 2024a). The distinction between NDEs and NDE-like experiences is therefore clinically and phenomenologically important, particularly when studies rely on self-reported experiences rather than medically verified events. In such cases, standardized assessment tools and careful qualitative interpretation are both needed to avoid overgeneralizing from heterogeneous subjective reports.

The occurrence and characteristics of NDEs have been investigated in both clinical and non-clinical populations. Prospective studies among cardiac arrest survivors and critically ill patients have reported that a minority of survivors describe NDEs after recovery, with incidence estimates varying according to population, setting, and measurement method (Parnia et al., 2001, 2023; van Lommel et al., 2001; Klemenc-Ketis et al., 2010; Rousseau et al., 2023). In one prospective study of survivors of prolonged critical illness, NDEs were reported by approximately 15% of participants, and psychological characteristics such as dissociative tendency and spiritual wellbeing appeared to be more closely associated with NDE occurrence than several medical parameters (Rousseau et al., 2023). Other studies have examined physiological factors such as carbon dioxide levels, cerebral hypoxia, ischemia, rapid eye movement (REM)-related mechanisms, and neurochemical changes as possible contributors to NDE phenomenology (Nelson et al., 2006; Klemenc-Ketis et al., 2010; Peinkhofer et al., 2019). Neurophysiological findings, including changes in brain activity near death or during syncope-induced unresponsiveness, further suggest that altered brain states may contribute to experiences later interpreted as NDEs or NDE-like experiences (Vicente et al., 2022; Xu et al., 2023; Martial et al., 2024b,^2025^).

However, neurobiological explanations alone cannot fully explain how individuals describe, interpret, and integrate these experiences. NDEs are also narrative and meaning-making events, shaped by memory, language, personal expectations, religious background, and cultural knowledge. A purely physiological account may explain some perceptual features, such as bright light, bodily detachment, or altered time perception, but it cannot by itself account for why specific figures, landscapes, moral themes, or afterlife images appear in particular narratives. For this reason, a growing body of research has emphasized the need to understand NDEs as experiences located at the intersection of neuropsychology, subjective consciousness, and cultural interpretation (Kellehear, 1993; Hashemi et al., 2023; Behzadi et al., 2025; Shushan, 2025).

Cross-cultural studies have shown that NDE reports often contain both recurrent and culturally variable elements. Features such as out-of-body experiences, peacefulness, encounters, light, boundary-crossing, and transitions to other realms have been reported in different cultural settings (Kellehear, 1993; Cassol et al., 2018; Hashemi et al., 2023; Shushan, 2025). At the same time, the symbolic content of these experiences may vary according to local religious traditions, afterlife beliefs, and culturally available imagery. In Western contexts, NDE narratives may include references to Christian figures, angels, heaven, or deceased relatives, whereas studies from Islamic, Buddhist, and other non-Western contexts have described different spiritual figures, moral landscapes, or cosmological structures (Kellehear, 1993; Murphy, 2001; Belanti et al., 2008; Hashemi et al., 2023; Behzadi et al., 2025; Shushan, 2025). These findings suggest that NDEs may involve a combination of relatively recurrent phenomenological features and culturally specific narrative forms. Culture should therefore not be treated as a superficial addition to NDE reports, but as a possible interpretive framework through which ambiguous and extraordinary experiences are described and made meaningful.

The Chinese cultural context provides an important setting for examining this issue. Chinese understandings of death and the afterlife are historically shaped by multiple traditions, including folk religion, Buddhism, Daoism, Confucian ethics, ancestor-related beliefs, karmic ideas, moral judgment, and popular images of the underworld. Motifs such as Guimen Pass, Black and White Impermanence, karmic judgment, and reincarnation are widely recognizable in Chinese cultural imagination, even among individuals who do not identify with a formal religion. Such motifs may provide symbolic resources through which some people describe experiences occurring near death or in altered states that resemble NDEs. Nevertheless, empirical research on NDEs in Chinese populations remains limited compared with studies from Western clinical contexts and several other cultural settings (Kellehear, 1993; Behzadi et al., 2025; Shushan, 2025). This gap restricts current understanding of how NDE phenomenology may be narrated and interpreted in East Asian cultural contexts.

Standardized psychometric instruments have played an important role in the development of NDE research. The Greyson Near-Death Experience Scale has long been used to identify and quantify core NDE features (Greyson, 1983). More recently, the Near-Death Experience Content (NDE-C) scale was developed to provide a broader assessment of NDE phenomenology, including dimensions such as transcendence of the ordinary, harmony, insight, border, and gateway (Martial et al., 2020b). The Chinese version of the NDE-C scale has also been translated and validated, providing a useful tool for assessing NDE content in Chinese-speaking populations (Li et al., 2024). These instruments allow researchers to compare the intensity and structure of experiences across participants and cultural contexts. However, standardized scale scores alone cannot fully capture culturally specific symbols, narrative organization, emotional interpretation, or the personal meanings attached to such experiences. Therefore, quantitative measurement needs to be complemented by qualitative analysis of participants’ own descriptions.

Qualitative research has contributed substantially to understanding the phenomenology of NDEs by identifying recurring themes such as out-of-body experience, altered perception, encounters, peacefulness, fear, life review, and ineffability (Cassol et al., 2018). Narrative-based approaches are particularly useful for examining how participants organize sensory impressions, emotions, and cultural references into meaningful accounts. They also allow researchers to distinguish between brief endorsement of scale items and richer narrative elaboration. At the same time, qualitative findings must be interpreted cautiously, especially when based on small, self-selected samples. The presence of culturally specific motifs in a limited number of narratives cannot establish population-level prevalence, causal mechanisms, or direct cultural determination of experience. Instead, such findings are best understood as exploratory evidence that may generate hypotheses for future studies using larger and clinically verified samples.

A mixed-methods design is therefore appropriate for studying self-reported NDEs and NDE-like experiences in an underexplored cultural context. Mixed-methods research can integrate standardized quantitative descriptions with qualitative examination of context, meaning, and culturally situated interpretations (Fetters et al., 2013). Accordingly, the present study combined the Chinese NDE-C scale with qualitative thematic analysis to examine both recurrent phenomenological features and culturally specific narrative content.

The present study should be understood as an exploratory investigation of self-reported NDEs and NDE-like experiences in a Chinese online convenience sample. Because recruitment was conducted through social media and the reported near-death events were not verified through medical records or third-party evidence, the study does not aim to establish clinically confirmed NDEs or to make representative claims about the Chinese population. Rather, it aims to provide a preliminary description of the phenomenological characteristics and narrative content reported by participants who described NDEs or NDE-like experiences. Particular attention is given to whether culturally recognizable Chinese motifs appear in these narratives and how quantitative NDE-C scores and qualitative themes may complement each other in describing the reported experiences. By adopting this cautious exploratory framing, the study seeks to contribute localized empirical material to cross-cultural NDE research while acknowledging the methodological limitations inherent in self-reported online data.

## 2 Materials and methods

### 2.1 Study design, participants, and ethics

This study employed an exploratory mixed-methods design to examine self-reported near-death experiences (NDEs) and near-death-like experiences in a Chinese online sample. Quantitative data were first collected using standardized questionnaires, followed by qualitative thematic analysis of open-ended narrative responses. The quantitative component was used to describe participants’ demographic characteristics, contextual information, and NDE-C scores, whereas the qualitative component was used to examine the phenomenological structure, subjective meanings, and culturally specific narrative content of the reported experiences. The two components were integrated at the interpretation stage by comparing quantitative group-level patterns with qualitative theme frequencies and narrative content. Because this study was conducted online and relied on retrospective self-report, participants’ medical records, physiological status at the time of the experience, and third-party verification were not available. Therefore, all classifications in this study should be understood as based on self-report and NDE-C responses rather than clinically verified NDE diagnoses.

Participants were recruited through purposive sampling via online social media platforms in China, including WeChat, Weibo, and Zhihu. Recruitment advertisements targeted individuals who reported having experienced near-death experiences or near-death-like experiences, including experiences triggered by illness, accidents, meditation, sleep-related states, or spiritual practices. Participants completed the survey through an online questionnaire platform. A total of 81 questionnaires were returned during data collection. The inclusion criteria were: (1) age 18–85 years; (2) self-reported experience of a near-death or near-death-like episode; and (3) provision of a valid response to the questionnaire. After screening incomplete or invalid questionnaires, 63 participants were included in the quantitative analysis. Among them, 48 participants provided complete narrative descriptions suitable for qualitative analysis, including 23 participants in the NDE group and 25 participants in the NDE-like group. Participants who reported an experience but scored below the NDE-C threshold were retained as a below-threshold comparison group for quantitative analysis but were not included in the qualitative thematic analysis. The participant flow from online survey collection to final inclusion in the quantitative and qualitative analyses is shown in Figure 1.

**FIGURE 1:**
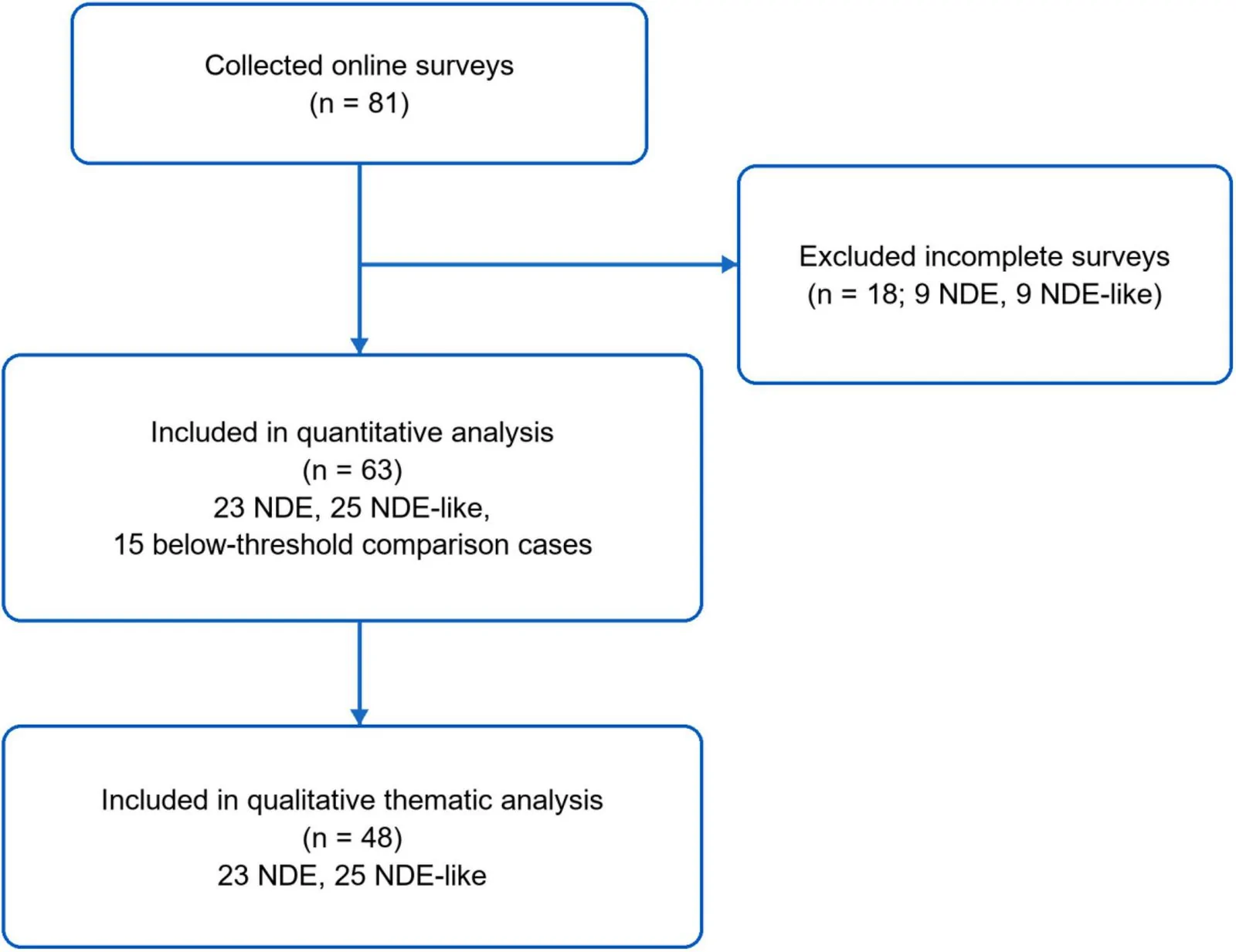
Flow diagram of online survey collection, screening, and final inclusion in the quantitative and qualitative analyses.

The first page of the online questionnaire presented the study objectives, confidentiality information, and informed consent statement. Participants could proceed only after selecting the “agree” option. The study protocol was approved by the Ethics Review Committee of the School of Basic Medical Sciences, Hangzhou Normal University (approval no. 20230322-4). The data were accessed for research purposes from 1 May 2023, to 1 April 2025. The original survey platform collected IP addresses and contact details for follow-up purposes. Before analysis, direct identifiers were removed, and participants were assigned anonymized study codes. Identifying information was not included in the analytic datasets used for qualitative coding, statistical analysis, or reporting. All data were stored securely and handled in accordance with the approved ethical protocol, and access was restricted to authorized research team members.

### 2.2 Measures and group classification

The online survey consisted of three components: sociodemographic and background information, the Chinese version of the Near-Death Experience Content (NDE-C) scale, and open-ended narrative responses. Sociodemographic and background information included sex, age, occupation, religious affiliation, marital status, educational attainment, self-rated health status, and contextual circumstances of the reported experience. Participants were also asked about the specific cause or trigger of the experience and whether they had used any substances that might alter consciousness around the time of the experience. The Chinese version of the NDE-C scale was used to assess the phenomenological content of the reported experiences. The NDE-C consists of 20 items rated on a Likert-type scale. Item scores are summed to yield a total score ranging from 0 to 80. Following established criteria, a total score of 27 or higher was used to identify experiences with salient NDE characteristics. This threshold was used for group classification rather than as an inclusion criterion for the entire quantitative analysis. To obtain subjective narrative data, participants were asked an open-ended question at the end of the survey: “Please describe your experience as fully as possible, including what you saw, smelled, felt, and went through.” These free-text responses were used for qualitative thematic analysis.

Participants were categorized into three groups for quantitative analysis based on both the reported context of the experience and NDE-C responses. Participants whose narratives described an actual or perceived life-threatening context and whose NDE-C scores met the threshold were classified as the NDE group. Participants whose experiences shared NDE-like phenomenological features but occurred in non-life-threatening, sleep-related, meditative, or otherwise ambiguous contexts were classified as the NDE-like group. Participants who reported an unusual experience but scored below the NDE-C threshold were classified as the below-threshold comparison group. Because clinical verification was not available, these categories should not be interpreted as clinically confirmed diagnostic groups. Instead, they represent self-reported experiential categories derived from participants’ descriptions and NDE-C responses. For brevity, these self-report-based categories are hereafter referred to as the NDE group, the NDE-like group, and the below-threshold comparison group.

### 2.3 Data analysis

Quantitative analyses were primarily descriptive. Sociodemographic characteristics, contextual information, trigger categories, and NDE-C total and item-level scores were summarized across the three groups. Continuous variables were summarized as means and standard deviations or medians and interquartile ranges, depending on distributional characteristics. Categorical variables were summarized as counts and percentages. Between-group comparisons were conducted using analysis of variance or non-parametric tests for continuous variables, and chi-square tests or Fisher’s exact tests for categorical variables, as appropriate. Effect sizes were reported where applicable. Because the groups were partly defined using NDE-C responses, group differences in NDE-C total scores were interpreted descriptively rather than as independent evidence of discriminant validity.

Qualitative analyses were conducted on 48 valid narrative accounts. The analysis was informed by Braun and Clarke’s six-phase approach to thematic analysis: familiarization with the data, generation of initial codes, searching for themes, reviewing themes, defining and naming themes, and writing the report (Braun and Clarke, 2006). The coding framework combined deductive and inductive approaches. Deductively, initial sensitizing concepts were informed by established NDE phenomenology, including out-of-body experiences, tunnel or transition imagery, perception of bright light, encounters with entities, panoramic life review, emotional valence, and long-term transformational effects. Inductively, additional themes were allowed to emerge from the Chinese-language narratives, especially culturally specific motifs that were not fully captured by existing NDE frameworks. Culturally specific Chinese imagery was treated as an emergent theme rather than an a priori category. This theme included explicit references to culturally recognizable Chinese Buddhist, Daoist, or folk-religious imagery related to death, afterlife realms, or cosmological transition, such as Guimen Pass, Black and White Impermanence, Lord Chenghuang, reincarnation, karmic judgment, and Chinese underworld-related imagery. Generic references to gods, spirits, light, or unidentified entities were not coded as culturally specific Chinese imagery unless they were explicitly embedded in a recognizable Chinese cultural context. Ambiguous descriptions were not assigned to this theme solely on the basis of the researchers’ interpretation.

All narratives were coded at the participant-theme level. Each theme was coded as present or absent in each participant’s narrative. Closely related expressions were consolidated into broader themes. For example, tunnel-like passage, cave imagery, passage through a tunnel, and falling through a passage were combined under “tunnel passage.” References to white light, bright light, light at the end of a passage, and a luminous world were combined under “bright light.” Entity-related subthemes were retained for descriptive purposes but were collapsed into the broader theme of “entities” in the clustering analysis to avoid parent-child duplication. The final thematic framework consisted of six higher-order domains: perceptual and bodily alteration, transitional and spatial imagery, encounter-related themes, cognitive and meaning-making themes, emotional valence, and culturally specific Chinese imagery.

Two researchers independently coded all 48 valid narrative accounts. Inter-coder reliability was first assessed at the participant-theme level and then summarized at the higher-order domain level, consistent with the organization of the qualitative results. For the six higher-order domains, percentage agreement ranged from 85.42% to 91.67%, and Cohen’s kappa ranged from 0.333 to 0.778. Except for the low-prevalence culturally specific Chinese imagery domain, domain-level kappa values ranged from 0.644 to 0.778. Domain-level reliability estimates are provided in Supplementary Table 1. Because several themes were rare or had imbalanced distributions, kappa values were interpreted cautiously and were considered alongside percentage agreement. After the initial reliability assessment, all coding discrepancies were reviewed against the original Chinese narratives. Discrepancies were resolved through discussion, and a final consensus coding matrix was generated. All theme frequencies, higher-order domain summaries, exploratory group comparisons, and clustering analyses reported below were based on this final consensus matrix.

Theme frequencies were summarized separately for the self-reported NDE and NDE-like groups as counts and percentages. Fisher’s exact tests were conducted for exploratory comparison of theme frequencies between groups, with false discovery rate (FDR) correction applied for multiple comparisons. The descriptive subtheme “other entities” was excluded from between-group testing to avoid duplication with the broader parent theme of “entities.” Given the small sample size and exploratory design, these tests were interpreted descriptively rather than as confirmatory evidence. The full results of these exploratory tests are provided in the Supplementary material. To explore relationships among themes, an exploratory theme co-occurrence analysis was conducted using the final participant-by-theme consensus matrix. Themes were represented as binary variables indicating whether each theme was present or absent in each narrative. Pairwise theme dissimilarity was calculated as one minus the Jaccard similarity coefficient. Jaccard distance was selected because it is appropriate for sparse binary qualitative data and does not treat shared absences as evidence of similarity. Hierarchical agglomerative clustering was then performed using average linkage. For the main analysis, clustering was conducted among self-reported NDE narratives only, because the purpose was to examine the internal thematic structure of NDE narratives. A secondary clustering analysis including all 48 NDE and NDE-like narratives was conducted as a supplementary analysis. The clustering analysis was used only as a descriptive visualization of theme co-occurrence patterns and was not interpreted as confirmatory evidence of causal relationships or population-level structure.

## 3 Results

### 3.1 Participant characteristics and qualitative sample

A total of 63 participants were included in the quantitative analysis, including 23 participants in the NDE group, 25 in the NDE-like group, and 15 in the below-threshold comparison group. The three groups were broadly comparable in sex, age, marital status, religious affiliation, self-rated health status, educational attainment, and substance use. NDE-C total scores differed markedly across groups, with the highest scores in the NDE group, followed by the NDE-like group and the below-threshold comparison group. Trigger categories also differed across groups, with near-drowning, head trauma, and anesthesia or surgery more common in the NDE group, whereas sleep-related triggers were concentrated in the NDE-like group. Because NDE-C responses contributed to group classification, differences in NDE-C scores should be interpreted descriptively rather than as independent evidence of discriminant validity. Participant characteristics and NDE-C scores are summarized in Table 1.

**TABLE 1:** Participant characteristics and NDE-C scores.

Variable	NDE, *n* = 23	NDE-like, *n* = 25	Below-threshold comparison, *n* = 15	*P*-value	Effect size
Sex				0.727	0.101
Male	10 (43.5%)	13 (52.0%)	6 (40.0%)	–	–
Female	13 (56.5%)	12 (48.0%)	9 (60.0%)	–	–
Age, mean ± SD	29.5 ± 7.4	28.9 ± 10.0	28.3 ± 10.4	0.924	0.003
Marital status				0.611	0.146
Married	10 (43.5%)	7 (28.0%)	6 (40.0%)	–	–
Unmarried	11 (47.8%)	16 (64.0%)	9 (60.0%)	–	–
Divorced	2 (8.7%)	2 (8.0%)	0 (0.0%)	–	–
Religious affiliation				0.861	0.069
Yes	4 (17.4%)	3 (12.0%)	2 (13.3%)	–	–
No	19 (82.6%)	22 (88.0%)	13 (86.7%)	–	–
Self-rated health status				0.285	0.242
Excellent	17 (73.9%)	16 (64.0%)	6 (40.0%)	–	–
Good	3 (13.0%)	7 (28.0%)	6 (40.0%)	–	–
Fair	1 (4.3%)	2 (8.0%)	2 (13.3%)	–	–
Poor	2 (8.7%)	0 (0.0%)	1 (6.7%)	–	–
Educational attainment				0.288	0.277
Junior high school	0 (0.0%)	2 (8.0%)	2 (13.3%)	–	–
High school	5 (21.7%)	3 (12.0%)	1 (6.7%)	–	–
College or vocational school	6 (26.1%)	5 (20.0%)	2 (13.3%)	–	–
Bachelor’s degree	12 (52.2%)	13 (52.0%)	7 (46.7%)	–	–
Master’s degree or above	0 (0.0%)	2 (8.0%)	3 (20.0%)	–	–
NDE-C total score, mean ± SD	47.9 ± 13.4	43.8 ± 11.1	16.5 ± 6.8	<0.001	0.570
Trigger				< 0.001	0.558
Cardiac arrest	0 (0.0%)	0 (0.0%)	1 (6.7%)	–	–
Near-drowning	6 (26.1%)	0 (0.0%)	1 (6.7%)	–	–
Head trauma	6 (26.1%)	0 (0.0%)	2 (13.3%)	–	–
Vascular accident	1 (4.3%)	0 (0.0%)	0 (0.0%)	–	–
Anesthesia or surgery	4 (17.4%)	0 (0.0%)	3 (20.0%)	–	–
Sleep-related	0 (0.0%)	10 (40.0%)	2 (13.3%)	–	–
Other medical	5 (21.7%)	9 (36.0%)	5 (33.3%)	–	–
Other non-medical	1 (4.3%)	6 (24.0%)	1 (6.7%)	–	–
Substance use				0.865	0.068
Yes	4 (17.4%)	5 (20.0%)	2 (13.3%)	–	–
No	19 (82.6%)	20 (80.0%)	13 (86.7%)	–	–

SD, standard deviation; NDE-C, Near-Death Experience Content scale. Values are presented as *n* (%) unless otherwise indicated. Effect sizes are η^2^ for continuous variables and Cramér’s V for categorical variables. Because some categorical variables had sparse cells, especially trigger categories, between-group comparisons should be interpreted cautiously. Because NDE-C responses contributed to group classification, group differences in NDE-C total scores are reported descriptively and should not be interpreted as independent validation of group status.

Among the 63 participants included in the quantitative analysis, 48 participants provided valid narrative descriptions of self-reported NDEs or NDE-like experiences and were included in the qualitative thematic analysis. This qualitative sample comprised 23 NDE narratives and 25 NDE-like narratives. Two coders independently coded all 48 narratives. Inter-coder reliability was adequate for an exploratory qualitative analysis. At the higher-order domain level, percentage agreement ranged from 85.42% to 91.67%. Cohen’s kappa values were 0.678 for perceptual and bodily alteration, 0.644 for transitional and spatial imagery, 0.778 for encounter-related themes, 0.647 for cognitive and meaning-making themes, and 0.689 for emotional valence. The culturally specific Chinese imagery domain showed lower kappa (κ = 0.333) despite 87.50% agreement, reflecting the low prevalence and boundary-sensitive nature of this emergent theme. Domain-level reliability estimates are summarized in Supplementary Table 1. All discrepancies were resolved through consensus review, and the final consensus matrix was used for subsequent analyses.

### 3.2 Thematic findings and representative excerpts

The final themes were organized into six higher-order domains: perceptual and bodily alteration, transitional and spatial imagery, encounter-related themes, cognitive and meaning-making themes, emotional valence, and culturally specific Chinese imagery. Perceptual and bodily alteration was the most common higher-order domain in both groups, appearing in 16 of 23 self-reported NDE narratives and 13 of 25 NDE-like narratives. Emotional valence was also common, appearing in 13 NDE narratives and 10 NDE-like narratives. Transitional and spatial imagery appeared in 10 NDE narratives and nine NDE-like narratives. Encounter-related themes were observed in eight NDE narratives and six NDE-like narratives. Cognitive and meaning-making themes showed a descriptively higher frequency in the NDE group, appearing in nine NDE narratives compared with four NDE-like narratives. Culturally specific Chinese imagery appeared in four NDE narratives and one NDE-like narrative.

Several themes showed descriptively higher frequencies in the NDE group than in the NDE-like group. Peaceful emotions were reported in 12 NDE narratives compared with five NDE-like narratives. Perception of bright light appeared in 10 NDE narratives and seven NDE-like narratives. Out-of-body experiences were also reported in 10 NDE narratives and seven NDE-like narratives. Encounter-related themes also showed a descriptively higher frequency in the NDE group: entities were coded in eight NDE narratives and six NDE-like narratives, while deceased loved ones or spiritual entities were identified in eight NDE narratives but were absent from the NDE-like group. Cognitive and meaning-making themes also showed descriptively higher frequencies in the NDE group. Panoramic life review appeared in seven NDE narratives and four NDE-like narratives. Personal insights were identified in five NDE narratives and were absent from the NDE-like group. Culturally specific Chinese imagery was identified in four of 23 self-reported NDE narratives and one of 25 NDE-like narratives. The NDE narratives included references to Black and White Impermanence, Guimen Pass, Lord Chenghuang, underworld judgment, and reincarnation. The single NDE-like narrative referred explicitly to experiencing one’s own reincarnation. Thus, culturally specific Chinese imagery was descriptively more frequent in the NDE group but was not absent from the NDE-like group. This group difference did not remain statistically significant after correction for multiple comparisons. Some themes were more common in the NDE-like group. Fantastical experiences appeared in 11 NDE-like narratives and seven NDE narratives. Negative emotions were also slightly more frequent in the NDE-like group. Theme frequencies are summarized in Table 2.

**TABLE 2:** Final qualitative themes by higher-order domain and group.

Higher-order domain	Theme	NDE, *n* = 23	NDE-like, *n* = 25
Culturally specific Chinese imagery	Chinese imagery	4 (17.4%)	1 (4.0%)
Cognitive and meaning-making themes	Long-term transformational effects	2 (8.7%)	0 (0.0%)
Cognitive and meaning-making themes	Personal insights	5 (21.7%)	0 (0.0%)
Cognitive and meaning-making themes	Panoramic life review	7 (30.4%)	4 (16.0%)
Transitional and spatial imagery	Bright light	10 (43.5%)	7 (28.0%)
Transitional and spatial imagery	Tunnel passage	6 (26.1%)	3 (12.0%)
Transitional and spatial imagery	Boundless space	1 (4.3%)	2 (8.0%)
Encounter-related themes	Entities	8 (34.8%)	6 (24.0%)
Encounter-related themes	Deceased loved ones or spiritual entities	8 (34.8%)	0 (0.0%)
Perceptual and bodily alteration	Out-of-body experiences	10 (43.5%)	7 (28.0%)
Perceptual and bodily alteration	Bodily sensations	10 (43.5%)	10 (40.0%)
Perceptual and bodily alteration	Time stopping	3 (13.0%)	1 (4.0%)
Perceptual and bodily alteration	Fantastical experiences	7 (30.4%)	11 (44.0%)
Emotional valence	Peaceful emotions	12 (52.2%)	5 (20.0%)
Emotional valence	Positive emotions	7 (30.4%)	5 (20.0%)
Emotional valence	Negative emotions	3 (13.0%)	5 (20.0%)

“Other entities” was retained during coding but is not shown in the main table to avoid duplication with the broader theme of “entities.” The full theme frequency table is provided in Supplementary Table 2. Group differences are descriptive unless otherwise stated. The difference in culturally specific Chinese imagery was not statistically significant after false discovery rate (FDR) correction.

Representative excerpts from self-reported NDE narratives are presented in Table 3 to illustrate how key themes appeared in narratives with relatively rich phenomenological content, particularly those involving culturally specific imagery and meaning-making. Following completion of the consensus coding process, the two coders jointly selected excerpts to illustrate the defining characteristics of the relevant themes and to provide sufficient narrative context. The excerpts were selected for illustrative purposes rather than on the basis of statistical typicality or frequency. Excerpts from all five narratives classified as containing culturally specific Chinese imagery are provided in Supplementary Table 4. The excerpts also illustrate how cultural, emotional, perceptual, and meaning-making themes could overlap within individual narratives.

**TABLE 3:** Representative excerpts from self-reported Near-death experience (NDE) narratives.

Theme	Representative excerpt
Chinese imagery	“After reaching the end of the tunnel, I entered a vast, white world. There was a dilapidated stone gate. I somehow knew that it was Guimen Pass, and I told myself that I had arrived there.”
Chinese imagery	“I expected Black and White Impermanence to come and take me away. I felt that the soul does not truly die but continues to exist in another realm between the worlds of yin and yang. All wrongdoing is recorded and reviewed at death, influencing the circumstances of one’s next reincarnation.”
Chinese imagery	“Two people would not let me enter. They said that Lord Chenghuang would not receive me.”
Out-of-body experiences	“My body was transparent and had no physical form. My vision floated upward and stopped at a fixed point.”
Out-of-body experiences	“At that moment, ‘I’ could move freely from this perspective. The perspective itself had no body; it was transparent, like what people often describe as a soul leaving the body.”
Bright light/tunnel passage	“After a long time, I finally noticed a white point of light far away. Although it was faint, it strongly attracted me in the darkness.”
Bright light/tunnel passage	“Then I entered a tunnel full of light.”
Peaceful emotions	“My mood was especially comfortable. There was no fear at all.”
Entities	“Many different people came to find me.”
Deceased loved ones or spiritual entities	“During this brief out-of-body experience, I met my deceased father and my pet, and we communicated spiritually.”
Panoramic life review	“Scenes from my life appeared before me like slides, showing past events from different perspectives.”
Fantastical experiences	“Some abstract patterns and indescribable scenes floated up.”

Quotations were translated from Chinese by the authors and lightly edited for readability without altering their substantive meaning. The excerpts are illustrative rather than exhaustive. Excerpts from all five consensus-coded narratives containing culturally specific Chinese imagery are provided in Supplementary Table 4.

### 3.3 Exploratory comparisons and theme co-occurrence clustering

Exploratory Fisher’s exact tests were conducted to compare theme frequencies between the self-reported NDE and NDE-like groups. Because of the small sample size and multiple comparisons, these analyses were interpreted cautiously. In exploratory comparisons, the most pronounced descriptive group difference was observed for deceased loved ones or spiritual entities, which appeared in eight NDE narratives and no NDE-like narratives. This difference remained significant after false discovery rate correction. Personal insights and peaceful emotions showed descriptive differences between groups, but these did not remain significant after correction. Full exploratory test results are provided in Supplementary Table 3.

Exploratory hierarchical clustering was conducted using Jaccard distance based on the final consensus coding matrix. Entity subthemes were collapsed into the broader theme of “entities” for this analysis to avoid parent-child duplication. The primary clustering analysis restricted to the 23 self-reported NDE narratives is shown in Figure 2.

**FIGURE 2:**
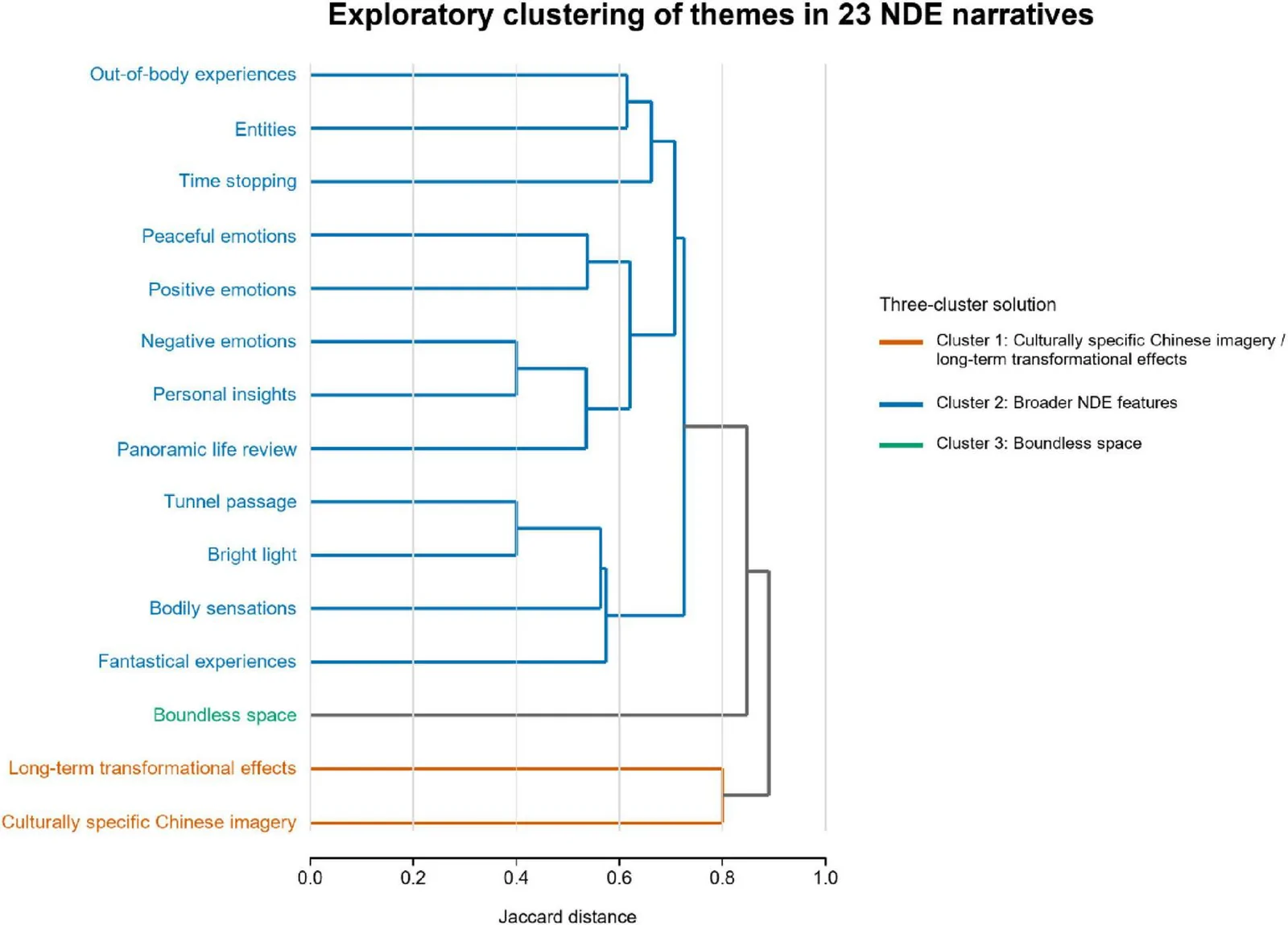
Primary exploratory clustering of themes in the 23 NDE narratives. Hierarchical clustering was performed using Jaccard distance based on the final participant-by-theme consensus matrix. Themes were coded as present or absent in each narrative, and average linkage was used. Colored theme labels indicate membership in the exploratory three-cluster solution. Entity subthemes were collapsed into the broader theme of entities to avoid parent-child duplication. The clustering is exploratory and descriptive.

In the three-cluster NDE-only solution, culturally specific Chinese imagery and long-term transformational effects were assigned to the same small cluster. However, both themes were infrequent, and their absolute Jaccard similarity was low. This cluster assignment should therefore be interpreted as a descriptive feature of the dendrogram rather than evidence of a reliable association or systematic co-occurrence. A broad second cluster contained most of the core experiential and affective features, including bright light, tunnel passage, entities, out-of-body experiences, bodily sensations, peaceful emotions, positive emotions, personal insights, negative emotions, panoramic life review, time stopping, and fantastical experiences. Within this broader cluster, bright light and tunnel passage appeared as a closely related pair, and peaceful emotions and positive emotions also showed close proximity. Boundless space appeared as a separate theme in the NDE-only clustering, reflecting its low frequency and limited co-occurrence with other themes.

A secondary clustering analysis including all 48 self-reported NDE and NDE-like narratives is presented separately in Supplementary Figure 1. These clustering results should be understood as exploratory descriptions of theme co-occurrence rather than evidence of causal or mechanistic relationships.

## 4 Discussion

### 4.1 Principal findings and overlap with previous research

This exploratory mixed-methods study examined self-reported NDEs and NDE-like experiences in a Chinese online sample using the Chinese NDE-C scale and qualitative narrative analysis. The findings showed that self-reported NDE and NDE-like narratives shared several recurrent phenomenological features, including out-of-body experiences, bright light, bodily sensations, emotional changes, and altered perceptual content. These themes are broadly consistent with established descriptions of NDE phenomenology, which commonly include out-of-body experiences, altered time perception, tunnel-like passage, bright light, encounters with deceased persons or other entities, life review, intense emotions, and changes in selfhood or meaning (Greyson, 1983; Charland-Verville et al., 2014; Cassol et al., 2018). Their presence in Chinese self-reported narratives suggests that several core NDE-related features may be phenomenologically recurrent across cultural contexts.

However, these features were not exclusive to the NDE group. Bright light, bodily sensations, out-of-body experiences, emotional changes, and fantastical experiences were also reported in NDE-like narratives. This overlap is consistent with previous research showing that NDE-like experiences can occur in non-life-threatening contexts, including syncope, meditation, sleep-related states, and other altered states of consciousness (Charland-Verville et al., 2014; Woollacott et al., 2024). Therefore, the presence of individual NDE-like features alone is not sufficient to establish a clinically verified NDE, especially in online self-report data.

At the same time, the NDE group showed a richer pattern of affective, encounter-related, and meaning-making themes. Peaceful emotions, life review, personal insights, and encounters with deceased loved ones or spiritual entities showed descriptively higher frequencies in the NDE group than in the NDE-like group. Culturally specific Chinese imagery was uncommon but theoretically informative, appearing in a small subset of narratives and showing a descriptively higher frequency in the NDE group. Except for encounters with deceased loved ones or spiritual entities, these descriptive differences did not remain statistically significant after correction for multiple comparisons. Together, these findings suggest that self-reported NDE and NDE-like experiences may overlap phenomenologically, while self-reported NDE narratives may involve more integrated emotional, relational, and existential meaning-making components.

### 4.2 Cross-cultural comparison and culturally specific Chinese imagery

One of the main contributions of this study is the identification of culturally specific Chinese imagery in self-reported NDE and NDE-like narratives. The specific motifs identified in the present narratives illustrate several culturally recognizable forms of Chinese afterlife imagery. In the present narratives, Black and White Impermanence were portrayed as figures associated with escorting the dead, Guimen Pass was described as a threshold to an afterlife realm, and Lord Chenghuang appeared in connection with entry into the underworld. References to the recording of wrongdoing, postmortem judgment, and the conditions of a subsequent rebirth can be interpreted in relation to historically established Chinese Buddhist and folk-religious conceptions of karmic retribution, underworld courts, and the passage between death and rebirth (Teiser, 2003; Wan-Sang et al., 2010; Graham, 2020). This interpretation concerns the cultural language through which the experience was narrated and does not imply that the reported imagery was caused exclusively by prior religious beliefs.

Comparison with studies conducted outside Asia indicates that several features identified in the present sample are not unique to Chinese narratives. Research involving predominantly Western participants has frequently described out-of-body experiences, bright light, tunnel-like transitions, encounters with deceased relatives or other entities, peaceful emotions, and life review as recurrent features of NDEs (Kellehear, 1993; Belanti et al., 2008; Shushan, 2025). The recurrence of these features in the present Chinese sample supports the possibility that some broad phenomenological components of NDEs are shared across cultural settings. Nevertheless, the symbolic identities assigned to encountered beings, the interpretation of transitional spaces, and the moral or religious meanings attributed to the experience may differ across societies. Western accounts, for example, may be narrated using images of heaven, angels, Christian figures, or reunion with deceased relatives, although substantial variation also exists within Western samples.

Studies from Asian and other non-Western settings provide a more direct comparison for the culturally specific content identified here. Thai NDE narratives have been discussed in relation to Buddhist beliefs, karmic consequences, locally recognizable religious figures, and culturally structured understandings of death and rebirth (Murphy, 2001). Indian NDEs have likewise been reported to contain culture-bound features and to differ in several respects from American cases (Pasricha and Stevenson, 1986). Comparisons of Japanese and Western NDEs have identified both shared features and differences in the interpretation of light, representations of the afterlife, and the occurrence of life review (Ohkado and Greyson, 2014). Related qualitative evidence from other non-Western contexts likewise suggests that NDE accounts may be interpreted through culturally available religious, relational, and moral frameworks (Hashemi et al., 2023; Behzadi et al., 2025). Cross-cultural research has likewise described how recurrent experiences such as light, encounters, boundary crossing, and movement toward another realm may be interpreted through locally available religious and cosmological frameworks (Shushan, 2025). In the present Chinese narratives, Guimen Pass, Black and White Impermanence, Lord Chenghuang, karmic judgement, reincarnation, and underworld courts may represent a comparable process of culturally situated narration. These motifs differ from the figures and afterlife settings commonly described in Western reports, but they perform broadly similar narrative functions by giving recognizable form to transitions, encounters, moral evaluation, and imagined postmortem destinations.

In the exploratory three-cluster NDE-only solution, culturally specific Chinese imagery and long-term transformational effects were assigned to the same small cluster. However, both themes were infrequent, and their absolute Jaccard similarity was low. The cluster assignment should therefore be understood as a descriptive feature of the dendrogram rather than evidence that culturally specific imagery was systematically associated with post-experience transformation. Taken together, these comparisons support an interpretation in which some phenomenological features may recur across cultural contexts, whereas their narrative expression is shaped by culturally available symbolic resources (Kellehear, 1993). However, the present study identified culturally specific Chinese imagery in only five narratives, and the data do not establish that prior cultural beliefs caused the reported experiences or that these motifs are representative of Chinese NDEs generally.

### 4.3 Methodological implications, limitations, and future directions

This study highlights the value of combining standardized assessment with qualitative narrative analysis. Standardized scales such as the Greyson NDE Scale and the NDE-C are useful for assessing the intensity and content of NDE-related experiences (Greyson, 1983; Martial et al., 2020b). The Chinese NDE-C provides an important tool for studying such experiences in Chinese-speaking populations (Li et al., 2024). However, scale scores alone cannot fully capture culturally specific symbols, narrative structure, emotional interpretation, or personal meaning. The present findings suggest that qualitative analysis can complement psychometric assessment by identifying culturally and narratively meaningful content that may not be fully represented in fixed response items.

Several limitations should be emphasized. First, this study was based on an online convenience sample recruited through social media. Participants were self-selected, and the sample may overrepresent individuals who were interested in unusual experiences, spirituality, or NDE-related topics. Therefore, the results cannot be generalized to the wider Chinese population. Second, the study relied on retrospective self-report. Medical records, physiological status, and third-party verification were not available. As a result, the study cannot determine whether participants experienced clinically verified NDEs during objectively life-threatening events. The categories used in this study should therefore be understood as self-reported NDE, NDE-like experience, and below-threshold comparison rather than clinically confirmed diagnostic groups.

Third, the sample size was small, particularly for qualitative subgroup comparisons, and culturally specific Chinese imagery occurred in only five narratives. Fourth, inter-coder reliability varied across themes. Although domain-level agreement was adequate for an exploratory qualitative analysis, some lower-level themes had lower kappa values because of low prevalence, overlapping definitions, or interpretive ambiguity. Future studies should refine the codebook, test it in larger and more diverse Chinese samples, and, where possible, recruit clinically verified participants from medical contexts such as cardiac arrest, intensive care, trauma, or coma recovery (Michael et al., 2025). Prospective designs would reduce recall bias and allow researchers to examine relationships between physiological variables, psychological traits, and later NDE reports (Parnia et al., 2001; van Lommel et al., 2001; Klemenc-Ketis et al., 2010; Rousseau et al., 2023). Further qualitative work should also examine whether motifs such as Guimen Pass, Black and White Impermanence, karmic judgment, reincarnation, and underworld imagery are associated with religious background, childhood exposure to folk beliefs, media influence, family narratives, or personal spirituality. Future cross-cultural research should use comparable sampling strategies, standardized NDE measures, and shared qualitative coding frameworks across Chinese, other Asian, and non-Asian populations to determine which features are phenomenologically recurrent and which are culturally specific.

## 5 Conclusion

This study provides empirical documentation of the coexistence of recurrent NDE-related features, such as out-of-body experiences, bright light, and peaceful emotions, with culturally recognizable Chinese imagery in a Chinese self-reported sample. The Chinese imagery included Guimen Pass, Black and White Impermanence, Lord Chenghuang, karmic judgement, reincarnation, and underworld courts. Although these motifs occurred in only five narratives, they are rarely represented in the predominantly Western NDE literature and are closely related to Chinese Buddhist and folk-religious traditions (Teiser, 2003; Wan-Sang et al., 2010; Graham, 2020). The central argument of this study is that culture is not merely a superficial addition to NDE narratives. Instead, it provides an important interpretive framework through which recurrent experiential features are organized, understood, and communicated (Kellehear, 1993; Shushan, 2025). This is important because neurobiological accounts may help explain how these experiences arise, but they cannot fully explain the culturally specific meanings attributed to them. The findings therefore support an integrative approach that considers biological processes together with psychological, social, and cultural meaning-making.

These findings may also have implications for culturally sensitive communication when individuals disclose such experiences in healthcare settings, although their clinical relevance requires further investigation in medically verified samples (Michael et al., 2025). Future studies should use clinically verified samples and timely interviews to determine whether these cultural motifs occur during the experience itself or emerge through subsequent recall and reconstruction.

## Data Availability

The dataset is not publicly available because it contains sensitive personal narrative information related to near-death and near-death-like experiences, which may compromise participant privacy. Aggregate and de-identified summary results are provided in the article and Supplementary material. Requests to access the datasets should be directed to Haibo Di, dihaibo19@aliyun.com.
