# Supplementary material for: Self-reported near-death and near-death-like experiences in a Chinese online sample: an exploratory mixed-methods study
Source: Front Psychol. 2026 Sep 21;17:1935246. doi: 10.3389/fpsyg.2026.1935246 (PMC13635375; doi:10.3389/fpsyg.2026.1935246)
Supplement: Supplementary file 1 [file Table_1.docx]

Supplementary Material

# 1 Supplementary Tables

## 1.1 Supplementary Table S1. Inter-coder reliability estimates at the thematic domain level

| **Higher-order domain** | **Percentage agreement** | **Cohen’s kappa** | **Interpretation** |
| --- | --- | --- | --- |
| Perceptual and bodily alteration | 85.42% | 0.678 | Adequate agreement |
| Transitional and spatial imagery | 85.42% | 0.644 | Adequate agreement |
| Encounter-related themes | 91.67% | 0.778 | Substantial agreement |
| Cognitive and meaning-making themes | 87.50% | 0.647 | Adequate agreement |
| Emotional valence | 85.42% | 0.689 | Adequate agreement |
| Culturally specific Chinese imagery | 87.50% | 0.333 | Lower kappa; interpreted cautiously because of low prevalence and boundary-sensitive coding |

**Note.** Reliability was calculated based on the initial independent coding of 48 valid narratives before consensus resolution. Percentage agreement and Cohen’s kappa were calculated at the higher-order thematic domain level. Kappa values were interpreted cautiously because several themes were rare or unevenly distributed across narratives. All coding discrepancies were subsequently reviewed against the original Chinese narratives and resolved through consensus discussion. The final consensus coding matrix was used for all theme frequency summaries, exploratory group comparisons, and clustering analyses reported in the manuscript.

## 1.2 Supplementary Table S2. Full aggregate theme frequency table by group

| **Higher-order domain** | **Theme** | **NDE, n = 23** | **NDE-like, n = 25** |
| --- | --- | --- | --- |
| Culturally specific Chinese imagery | Chinese imagery | 4 (17.4%) | 1 (4.0%) |
| Cognitive and meaning-making themes | Long-term transformational effects | 2 (8.7%) | 0 (0.0%) |
| Cognitive and meaning-making themes | Personal insights | 5 (21.7%) | 0 (0.0%) |
| Cognitive and meaning-making themes | Panoramic life review | 7 (30.4%) | 4 (16.0%) |
| Transitional and spatial imagery | Bright light | 10 (43.5%) | 7 (28.0%) |
| Transitional and spatial imagery | Tunnel passage | 6 (26.1%) | 3 (12.0%) |
| Transitional and spatial imagery | Boundless space | 1 (4.3%) | 2 (8.0%) |
| Encounter-related themes | Entities | 8 (34.8%) | 6 (24.0%) |
| Encounter-related themes | Deceased loved ones or spiritual entities | 8 (34.8%) | 0 (0.0%) |
| Encounter-related themes | Other entities | 0 (0.0%) | 6 (24.0%) |
| Perceptual and bodily alteration | Out-of-body experiences | 10 (43.5%) | 7 (28.0%) |
| Perceptual and bodily alteration | Bodily sensations | 10 (43.5%) | 10 (40.0%) |
| Perceptual and bodily alteration | Time stopping | 3 (13.0%) | 1 (4.0%) |
| Perceptual and bodily alteration | Fantastical experiences | 7 (30.4%) | 11 (44.0%) |
| Emotional valence | Peaceful emotions | 12 (52.2%) | 5 (20.0%) |
| Emotional valence | Positive emotions | 7 (30.4%) | 5 (20.0%) |
| Emotional valence | Negative emotions | 3 (13.0%) | 5 (20.0%) |

**Note.** Values are presented as n (%). Themes were coded as present or absent at the participant-theme level. The broader theme “entities” was retained to summarize encounter-related content, whereas “deceased loved ones or spiritual entities” and “other entities” were retained as descriptive subthemes. Because the broader and more specific entity-related themes may overlap conceptually, they should not be summed. No raw narrative data or participant-level qualitative transcripts are included in this table.

## 1.3 Supplementary Table S3. Exploratory Fisher’s exact test results for theme frequency comparisons

| **Theme** | **NDE, n = 23** | **NDE-like, n = 25** | **Fisher’s exact p value** | **FDR-adjusted p value** |
| --- | --- | --- | --- | --- |
| Deceased loved ones or spiritual entities | 8 (34.8%) | 0 (0.0%) | 0.001 | 0.021 |
| Personal insights | 5 (21.7%) | 0 (0.0%) | 0.020 | 0.157 |
| Peaceful emotions | 12 (52.2%) | 5 (20.0%) | 0.034 | 0.180 |
| Chinese imagery | 4 (17.4%) | 1 (4.0%) | 0.180 | 0.557 |
| Long-term transformational effects | 2 (8.7%) | 0 (0.0%) | 0.224 | 0.557 |
| Tunnel passage | 6 (26.1%) | 3 (12.0%) | 0.279 | 0.557 |
| Panoramic life review | 7 (30.4%) | 4 (16.0%) | 0.311 | 0.557 |
| Time stopping | 3 (13.0%) | 1 (4.0%) | 0.338 | 0.557 |
| Bright light | 10 (43.5%) | 7 (28.0%) | 0.367 | 0.557 |
| Out-of-body experiences | 10 (43.5%) | 7 (28.0%) | 0.367 | 0.557 |
| Fantastical experiences | 7 (30.4%) | 11 (44.0%) | 0.383 | 0.557 |
| Positive emotions | 7 (30.4%) | 5 (20.0%) | 0.511 | 0.651 |
| Entities | 8 (34.8%) | 6 (24.0%) | 0.529 | 0.651 |
| Negative emotions | 3 (13.0%) | 5 (20.0%) | 0.703 | 0.804 |
| Boundless space | 1 (4.3%) | 2 (8.0%) | 1.000 | 1.000 |
| Bodily sensations | 10 (43.5%) | 10 (40.0%) | 1.000 | 1.000 |

**Note.** Fisher’s exact tests were conducted as exploratory comparisons of theme frequencies between the self-reported NDE and NDE-like groups. False discovery rate correction was applied for multiple comparisons. Because of the small sample size, sparse cells, and exploratory study design, these results should be interpreted descriptively rather than as confirmatory evidence of group differences.

## 1.4 Supplementary Table S4. Culturally specific Chinese imagery identified in the final consensus narratives

| **Participant ID** | **Group** | **Cultural motif** | **Representative excerpt** |
| --- | --- | --- | --- |
| 25 | NDE | Black and White Impermanence | “I saw Black and White Impermanence.” |
| 34 | NDE | Guimen Pass | “There was a dilapidated stone gate. I somehow knew that it was Guimen Pass, and I told myself that I had arrived at Guimen Pass.” |
| 62 | NDE | Lord Chenghuang | “Two people would not let me enter. They said that Lord Chenghuang would not receive me.” |
| 68 | NDE | Black and White Impermanence; underworld judgement; reincarnation | “I expected Black and White Impermanence to come and take me away. I felt that the soul does not truly die but continues to exist in another realm between the worlds of yin and yang. All wrongdoing is recorded and reviewed at death, influencing the circumstances of one’s next reincarnation.” |
| 81 | NDE-like | Reincarnation | “I saw my own reincarnation.” |

**Note.** This table presents all five narratives classified as containing culturally specific Chinese imagery in the final consensus coding matrix. Quotations were translated from Chinese by the authors and abridged where necessary for readability. Participant numbers are anonymized study codes.

# 2 Supplementary Figures

## 2.1 Supplementary Figure S1. Secondary exploratory clustering of themes across all 48 NDE and NDE-like narratives

**Supplementary Figure S1.** Secondary exploratory clustering of themes across all 48 NDE and NDE-like narratives. Hierarchical clustering was performed using Jaccard distance based on the final participant-by-theme consensus coding matrix. Themes were coded as present or absent in each narrative, and average linkage was used. Colors indicate the exploratory three-cluster solution. Entity subthemes were collapsed into the broader theme of entities to avoid parent-child duplication. This secondary analysis is exploratory and descriptive and should not be interpreted as evidence of reliable associations, causal relationships, or population-level thematic structure.
